# Encoding of Temporal Information by Timing, Rate, and Place in Cat Auditory Cortex

**DOI:** 10.1371/journal.pone.0011531

**Published:** 2010-07-19

**Authors:** Kazuo Imaizumi, Nicholas J. Priebe, Tatyana O. Sharpee, Steven W. Cheung, Christoph E. Schreiner

**Affiliations:** 1 Coleman Memorial Laboratory, W. M. Keck Center for Integrative Neuroscience, Department of Otolaryngology-Head and Neck Surgery, University of California San Francisco, San Francisco, California, United States of America; 2 Neuroscience Center, Louisiana State University Health Sciences Center, New Orleans, Louisiana, United States of America; 3 Section of Neurobiology, School of Biological Sciences, University of Texas at Austin, Austin, Texas, United States of America; 4 Sloan-Swartz Center for Theoretical Neurobiology, University of California San Francisco, San Francisco, California, United States of America; 5 Computational Neurobiology Laboratory, Salk Institute for Biological Studies, La Jolla, California, United States of America; Hotchkiss Brain Institute, University of Calgary, Canada

## Abstract

A central goal in auditory neuroscience is to understand the neural coding of species-specific communication and human speech sounds. Low-rate repetitive sounds are elemental features of communication sounds, and core auditory cortical regions have been implicated in processing these information-bearing elements. Repetitive sounds could be encoded by at least three neural response properties: 1) the event-locked spike-timing precision, 2) the mean firing rate, and 3) the interspike interval (ISI). To determine how well these response aspects capture information about the repetition rate stimulus, we measured local group responses of cortical neurons in cat anterior auditory field (AAF) to click trains and calculated their mutual information based on these different codes. ISIs of the multiunit responses carried substantially higher information about low repetition rates than either spike-timing precision or firing rate. Combining firing rate and ISI codes was synergistic and captured modestly more repetition information. Spatial distribution analyses showed distinct local clustering properties for each encoding scheme for repetition information indicative of a place code. Diversity in local processing emphasis and distribution of different repetition rate codes across AAF may give rise to concurrent feed-forward processing streams that contribute differently to higher-order sound analysis.

## Introduction

An ultimate goal in auditory neuroscience is to understand the neural coding of species-specific communication and human speech sounds, but the complexity of such sounds renders this challenge difficult. A common approach is to reduce intractable experimental questions to tractable ones by studying key coding features using parametric techniques. Periodic amplitude modulations are ubiquitous temporal features of species-specific communication and human speech sounds [1], [2]. The modulation envelope of vocalization and speech (e.g., phonemes) is dominated by low repetition rates (<40 Hz) [2]–[5] and most cortical neurons limit their timing-locked responses to that modulation range [6]. Speech and vocalization decoding depends strongly on the integrity of the low rate repetition modulation envelope [7]–[10]. Lesion studies in monkeys and humans have suggested that auditory cortex (AC) is necessary to process communication or speech sounds [11], [12]. It has been proposed that precise spike timing may code slow repetition sounds, while firing rate (FR) may code faster repetition sounds in AC [6], [13]–[17] but see Ref. [18]. A recent study in marmoset monkeys proposed that FR may code a particular range of slow to medium repetition rates (∼10 to 45 Hz) in the anterior field of AC [19]. A growing number of studies suggest that interspike interval (ISI) profiles are a viable neural code for temporal processing [20]–[23]. However, ISI analysis of AC response patterns is not yet well advanced. A particular issue is that spike-timing precision and FR are not completely independent measures. Both bear on the potential efficacy of an interval code. We investigated stimulus-related neural information of spike-timing precision, FR, and ISIs for coding slow repetition rates and their topographic organization by high-resolution multi-unit mapping of a primary auditory field in the ketamine-anesthetized cat. This approach should be able to clarify the roles of timing and place codes in conveying information about low stimulus repetition rates.

Temporal information by spike timing and FR often appears to be spatially distributed in AC [3], [4], [24]. Organized spatial distributions (‘maps’) of these properties may provide an opportunity to explore how temporal information is represented by a population of cortical neurons [25]. In the cat, two tonotopic fields comprise the primary core areas at a hierarchically equivalent level, primary AC (AI) and anterior auditory field (AAF) [26], [27]. They receive largely independent, concurrent inputs from the different thalamic divisions [28], [29] resulting in different distributions of spectral receptive field parameters [30], [31]. Behavioral experiments with reversible cryoloop lesions suggest that cat AAF contributes to temporal-pattern discrimination [32] but is not involved in other functional tasks, such as sound localization [33]. This supports the notion that AAF is part of a stimulus identification or ‘what’ pathway [34].

Time-locking in AAF has been shown in several species to cover a wider frequency range than in other cortical fields [6], [35], [36], although the range is still dominated by modulation rates <∼40 Hz. This provides a comparatively wide repetition rate range to compare properties of phase locking, FR, and interval encoding of temporal information. Click trains are used to explore the encoding of repetitive stimuli in AAF. In contrast to sinusoidally amplitude-modulated signals [6], [25], [37], changes in click train repetition rates are not confounded by changes in stimulus rise times [38]. Here, we investigate different neural encoding schemes of slow repetition rate sounds and their spatially arranged expressions of stimulus-related mutual information.

## Results

To understand neural coding of slow repetitive sounds in AC, we obtained repetition rate transfer functions (RRTFs) to quantify responses to click trains. A population code is assumed and no distinction is made between local multi-unit and single-unit responses. We employed a high-resolution cortical mapping technique with extracellular recordings [30], [39] and reconstructed spatial organization via Voronoi-Dirichlet tessellation maps.

RRTFs were examined for 276 multi-unit recordings in cat AAF of three hemispheres (two left and one right). AAF is located anterior to AI and usually flanked by suprasylvian and anterior ectosylvian sulci [26], [28]. There was no clear evidence of a temporal coding difference between left and right hemispheres and they were treated equally in the population analyses.

### Measures of Vector Strength, Firing Rate, and Interspike Intervals

**Figure 1:**
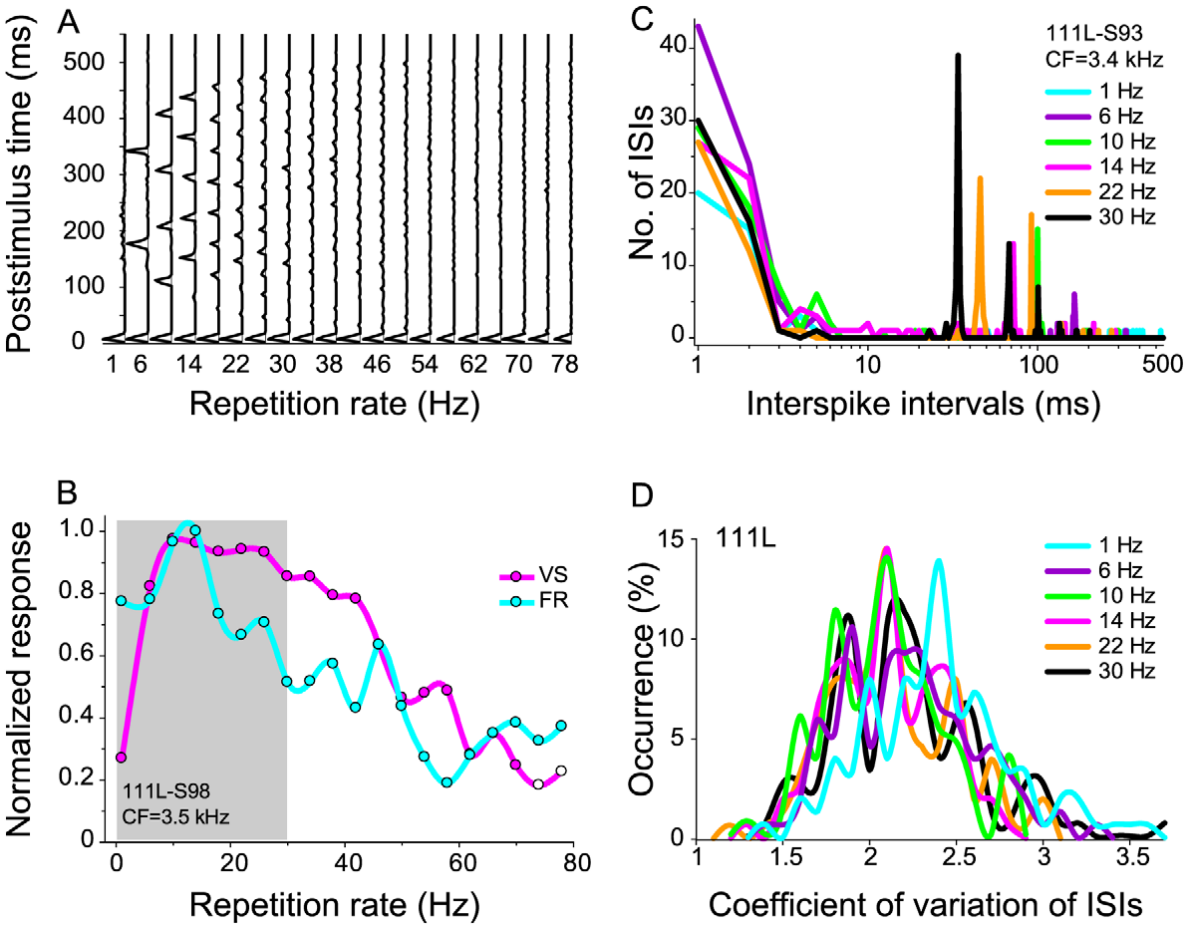
Repetition rate transfer functions for VS and FR. (A) Poststimulus time histograms for 20 repetition rates for an AAF site. Response strength was normalized to the maximum response at 1 Hz. Maximum height of the FR ordinate: 15 spikes. Information values for 111L-S98: VS info: 0.62 bits/stimulus; FR info: 0.23 bits/stimulus; ISI (1 ms) info: 0.51 bits/stimulus; ISI (10 ms) info: 1.95 bits/stimulus. (B) Corresponding RRTFs for VS (magenta line) and FR (blue line). Data points are fit by a polynomial cubic spline. Filled circles are significant VS values (Rayleigh test, *p*<0.001). Gray background illustrates the repetition rate range at the focus in this study. (C) ISI histogram for 6 repetition rates. Multiple ISI peaks correspond to integer multiples of stimulus periods. Information values for 111L-S93: VS info: 0.09 bits/stimulus; FR info: 0.11 bits/stimulus; ISI (1 ms) info: 1.20 bits/stimulus; ISI (10 ms): 2.28 bits/stimulus. (D) Population distribution of the coefficient of variation (CV) of ISIs for hemisphere 111L (*n* = 130). CV of ISI that estimates the variability of ISIs was computed by dividing the standard deviation of ISIs by the mean. Histograms of CV distributions, smoothed by a polynomial cubic spline, are illustrated for six different repetition-rates.

For RRTFs, two different measures have been used to describe temporal tuning. Spike-timing precision is expressed as vector strength (VS) measuring how well spikes are synchronized to the click stimulus relative to the duration of the repetition period (see Materials and Methods). VS values range from zero (spikes evenly distributed throughout the stimulus period) to one (spikes are perfectly aligned to a particular phase of the stimulus period). The other measure is average FR magnitude. Stimuli with low- and high-repetition rates may be coded differently by VS and FR [14]–[17]. Multi-unit examples of post-stimulus time histograms (PSTHs) for clicks at different repetition rates (1–38 Hz for many recording sites but up to 250 Hz presented 15 times; see Materials and Methods) reveal different response behaviors for VS and FR measures (Figs. 1A, S1A, S1B). Phase locking to the stimuli is expressed at varying degrees, with maximal values predominantly seen at low repetition rates. The corresponding RRTFs for the two measures, VS (magenta lines) and FR (cyan lines), show band-pass behavior (Fig. 1B). For a majority of recording sites, VS peaked at low repetition rates and declined with increasing (mid to high) repetition rates (spikes losing synchronization to the stimulus). Similar behavior was seen for FR (Fig. 1B). However, for other sites, FR often peaked at higher repetition rates than VS (Fig. S1C, S1D). On the average, FR peaked at 29.2±22.2 Hz (hereafter, expressed as mean ± standard deviation in the text), which was significantly higher than VS (12.8±8.1 Hz) (paired *t*-test; *p*<0.0001). This study was focused on encoding of low repetition rates (1, 6, 10, 14, 22, and 30 Hz), a range associated with the occurrence frequency of vocalization phrases, phonemes, or syllables (gray background in Figs. 1B, S1C, S1D) and with a high probability of encountering high temporal response fidelity.

Unlike VS and FR, ISI behavior has not been extensively studied in AC. Recent work, however, demonstrated that ISIs can present a sensible neural code for temporal processing [22], [40]. The ISI distribution for a cortical recording site in response to a range of low repetition rates is illustrated in Figure 1C. Some recording sites (<4%) only showed ISIs at short intervals of 1 to 3 ms, compatible with bursting, but not at the intervals corresponding to the period of the presented repetition rate (Fig. S2). A more common occurrence is recording sites that express ISI peaks corresponding to integer multiples of stimulus intervals (stimulus phase-locked spikes) (Fig. 1C). Unlike VS and FR, ISI is not directly characterized by a single value. The coefficient of variation (CV), an estimate of ISI variability computed by dividing the ISI standard deviation by the mean, is the main descriptor of the ISI distribution. Figure 1D illustrates population histograms of CV of ISIs for six difference repetition rates for all recording sites in one hemisphere. The CV distributions of ISIs were quite similar across repetition rates. However, medium repetition rates differed slightly from 1 and 30 Hz (*p*<0.05; Fisher's protected least significant difference for a multiple *t*-statistics indicating higher ISI fidelity for that range). The CV of ISIs spanned from ∼1 to >3, which was higher than values derived from visual cortex (<∼1) [41], [42], although differences in the auditory and visual stimulus paradigms make a direct comparison of the values difficult.

### Neural ISI Code for Low Repetition Rates

The three response measures carried different amounts of information about the temporal stimulus properties. To quantify the information content, i.e., estimating the ability to discriminate between different repetition rates based on their cortical response, we considered the unit-basis mutual information (MI), as read out by an ideal observer (see Materials and Methods).

The MI for ISI was calculated for two conditions: ISI (1 ms) was based on all intervals ≥1 ms, whereas ISI (10 ms) was based only on intervals ≥10 ms, more closely matching the interval range contained in the presented repetition range. The different repetition rates can be distinguished only for ISI values ≥10 ms (cf. Fig. 1C). Shorter ISIs (<3ms) occurred most often, but they did not allow distinguishing between different information values of the presented repetition rate range. For the example site in Figure 1C, when we included only ISI values ≥10 ms, we obtained an information value of ∼2 bits (out of a maximum of ∼2.58 bits ( = log_2_(6)) for comparing six stimuli). This value is consistent with the visual inspection of Figure 1C, where four of six repetition rates (10 Hz, 14 Hz, 22 Hz, and 30 Hz) can be reliably distinguished. At an ISI resolution of 1 ms, the information for the example is somewhat reduced (∼1.5 bits/stimulus)

**Figure 2:**
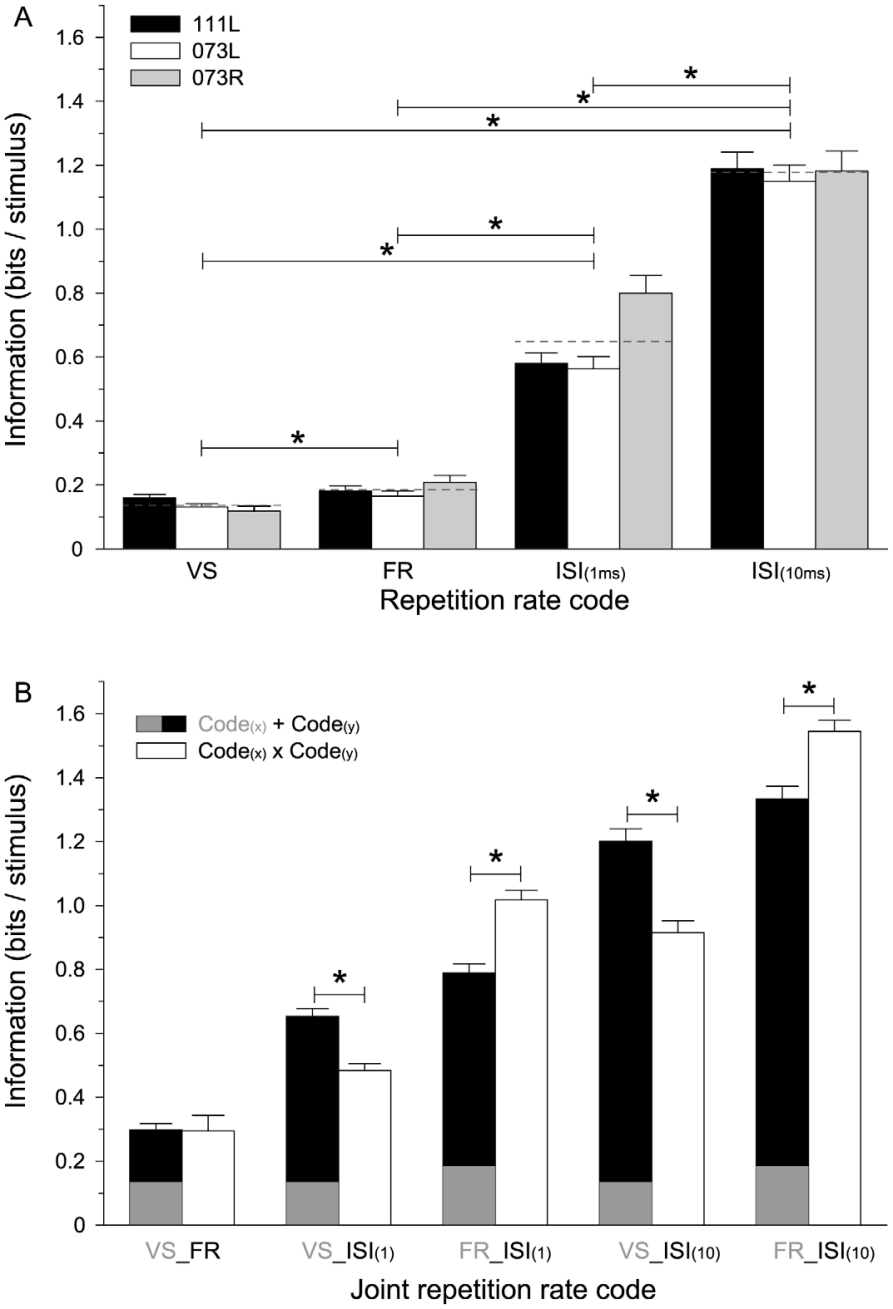
Mutual information (MI) contained in VS, FR, and ISI. (A) Mean (± standard error of the mean) of MI values for VS, FR, ISI (1 ms) and ISI (10 ms) for all three hemispheres. The global mean is indicated as a dashed line across the three hemispheres. MI for ISI (10 ms) was based on intervals equal or larger than 10 ms, whereas MI for ISI (1 ms) contained all intervals equal or larger than 1 ms. Paired *t* tests adjusted by the sequential Bonferroni correction for multiple comparisons (*p*<0.001) were performed for the three global mean measures. A theoretical MI value for distinguishing six different repetition rate stimuli is 2.58 bits/stimulus ( = log_2_(6)). (B) Information captured for different combinations of a joint repetition rate code. Black/gray bars: additive combination of the two codes (Code_(x)_ + Code_(y)_). The number of sites that resulted in valid joint information value was lower than the total number of sites for the individual information analysis. The summed information is based on recording sites that had a valid joint information. White bars: joint information values for two codes (Code_(x)_ × Code_(y)_). Unpaired *t* tests for the comparison between additive and joint codes (*p*<0.001).

MI contained in ISI (hereafter, ISI information) was significantly higher than that contained in either VS or FR (hereafter, VS and FR information, respectively) (*p*<0.001; paired *t* tests adjusted by the sequential Bonferroni correction for multiple comparisons; Fig. 2A). Across all hemispheres, ISI (1ms) information averaged to 0.63±0.40 bits/stimulus compared to 0.14±0.12 for VS information and 0.18±0.18 for FR information. ISI (10ms) information was almost twice as high (1.18±0.54), reflecting the reduction of short-interval noise. ISI (10ms) information was highly correlated with ISI (1ms) information (*r^2^* = 0.66; *p*<0.001).

To assess whether combining the different encoding schemes can capture an increased amount of repetition information over each individual scheme, we tested combinations of encoding pairs for all recording sites that resulted in a significant amount of information for the joint schemes (Fig. 2B). Analysis of a combination including all three schemes failed due to an insufficient number of appropriate sites.

Joint information estimates equal to the sum of information by the individual schemes would indicate non-redundancy of the contributing information. Combining VS and FR information (Fig. 2B, white bars) was almost equal to the sum of information by both individual schemes (Fig. 2B, black/gray bars), suggesting that each carried non-redundant information. Furthermore, combining FR and ISI (1ms or 10ms) codes resulted in an increase of information beyond the linear sum for the individual schemes. This behavior is indicative of non-redundant contributions of each scheme for encoding repetition information with a cooperative, synergistic component for the FR and ISI combination. By contrast, VS combined with ISI information resulted in lower information than the sum, indicating that both schemes conveyed redundant information (Fig. 2B).

**Figure 3:**
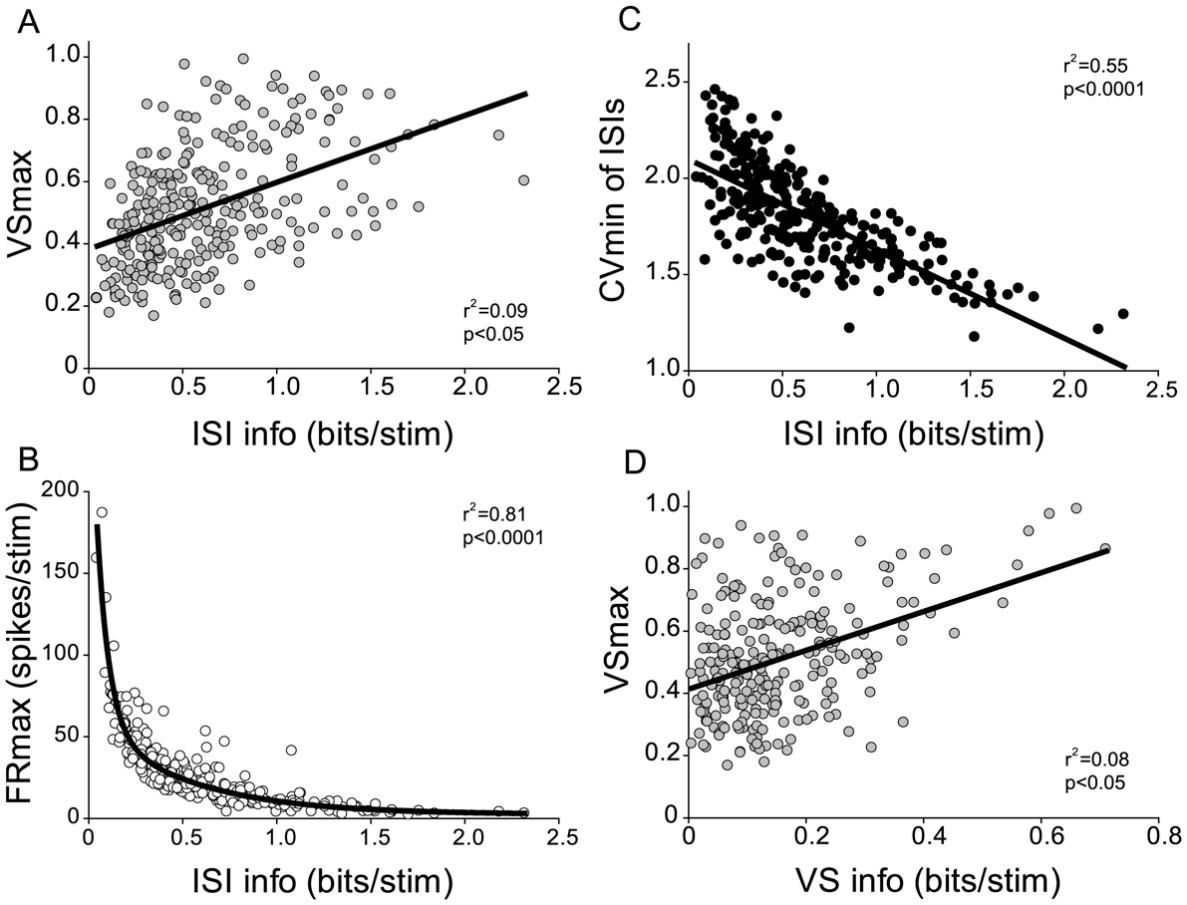
Correlation between information and three temporal response measures. (A) Positive correlation between VS max and ISI info (*p*<0.05). (B) Negative exponential correlation between ISI info and FR max (*p*<0.0001). (C) Negative correlation between ISI info and CV min (*p*<0.0001). (D) Weak positive correlation between VS info and VS max (*p*<0.05).

ISI information correlated with the magnitude of all three response measures, VS, FR, and CV of ISIs (Fig. 3). The rest of the analysis will focus on the ISI information at 1 ms resolution since it does not assume *a priori* knowledge of the stimulus periodicity range. The maximum value evoked by any of the tested repetition rates was used to represent overall spike-timing precision (VS max) and FR magnitude (FR max) for each recording site. ISI precision is represented by the minimum coefficient of variation (CV min) of ISI. The strongest correlation existed between FR max and ISI information: the lower FR, the higher was the ISI information (Fig. 3B). CV min was negatively correlated with ISI information: the less ISI variability, the higher was the ISI information (Fig. 3C). VS max was weakly positively correlated with ISI information (Fig. 3A). All three measures contributed to ISI information indicating that temporal coding is not dominated by a single response aspect. VS information was weakly but significantly correlated with VS max: sites with high VS max values carry more VS information (Fig. 3D). By contrast, FR magnitude is not significantly correlated with FR information (data not shown).

Although the higher ISI information relative to VS and FR information suggested that discrimination between low repetition rates may be ISI dominated in cat AAF, the other forms of stimulus encoding still may be useful, especially if the three codes are either independently distributed or provide non-redundant information as demonstrated above for the joint information. Principal component analysis jointly applied to the three information estimates, the underlying response measures (VS max, FR max and CV min), as well as four additional receptive field parameters (characteristic frequency (CF), Q40, threshold and minimum latency, see Materials and Methods) revealed three orthogonal components of temporal processing (Table 1). As expected from the high ISI information values, the strongest factor, representing 28.6% of the variance across all three hemispheres, captured ISI information as well as the covariants of FR max and CV min. The second factor (17.0% of variance) was aligned with VS information and VS magnitude. The fifth factor (9.0% of variance) was dominated by repetition rate information carried by FR. This analysis indicates that the three schemes of repetition rate information carrier fall along orthogonal axes. However, it does not imply that they are completely independent from each other, as already shown by the joint information analysis. They indicate, however, that some of these aspects capture largely uncorrelated, non-redundant aspects about the repetition rates. The temporal factors were not correlated with the two factors comprising the spectral parameters, CF and Q40 (F3; 12.9%) and threshold and response latency (F4; 10.0%) (Table 1; see Ref. [30]). The temporal encoding schemes captured by the three orthogonal temporal factors provide alternative, though not completely independent, means of extraction, representation, and transmission of low repetition rate information.

**Table 1:** Principal component analysis.

Parameter	Factor 1	Factor 2	Factor 3	Factor 4	Factor 5
Explained Variance (%)	28.6	17.0	12.9	10.0	9.0
VS max	0.040	**0.907**	−0.080	−0.011	0.000
FR max	**0.794**	−0.089	−0.154	−0.196	0.326
CV min	**0.838**	0.191	0.009	−0.046	−0.327
VS info	0.204	**0.721**	−0.111	−0.125	0.348
FR info	−0.016	0.203	−0.069	0.016	**0.893**
ISI info	**−0.902**	−0.157	0.092	0.099	−0.023
CF	0.079	−0.067	**0.859**	−0.012	−0.115
Q40	−0.291	−0.102	**0.759**	−0.051	0.027
Threshold	−0.127	−0.023	−0.077	**0.801**	0.271
Latency	−0.101	−0.081	0.010	**0.774**	−0.255

Three click train response parameters (VS max, maximum vector strength; FR max, maximum firing rate; CV min, minimum coefficient of variations for ISIs), three mutual information values (VS info; FR info, ISI info), and four basic receptive field parameters (CF, characteristic frequency; Q40, sharpness of tuning; Threshold, response threshold; Latency, minimum latency) were analyzed. Analysis was applied jointly to all sites of the three hemispheres. Total variance accounted for: 77.5%. Bold numbers: dominant factor loadings for each parameter. Five significant factors were identified (Bartlett's chi-square test; *p*<0.05).

### Spatial Distribution of Low Repetition Rate Codes

**Figure 4:**
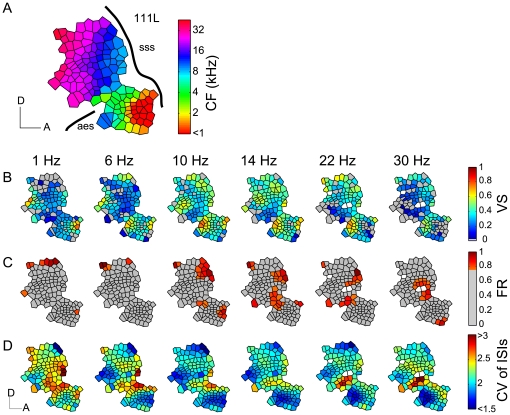
Spatial distribution of population response to different repetition rates. (A) Tonotopic gradient smoothed by a weighted least-squares linear regression model is reconstructed on the cortical surface by Voronoi-Dirichlet tessellation. An approximate location of AAF is indicated by the suprasylvian sulcus (sss) and the anterior ectosylvian sulcus (aes; thick black lines). Hemisphere 111L; D: dorsal, A: anterior, scale bars: 1 mm. (B) Spatial representation of VS as a function of different repetition rate. Repetition rates are shown on the top. White polygons indicate sites not tested for the corresponding repetition rates, which also apply to (C, D). Raw VS values of the Voronoi-Dirichlet tessellation maps were smoothed by a weighted least-squares linear regression model. (C) Spatial distribution of FR as a function of repetition rate. FR magnitude was normalized to the peak rate for the corresponding repetition rate. Normalized FR magnitudes were smoothed by a weighted least-squares linear regression model. High activity sites with FR>0.75 in the smoothed maps were categorized and shown in the bottom panel as red polygons. Sites with FR<0.75 are illustrated by gray polygons. (D) Spatial distribution of CV of ISI as a function of repetition rate. Spatially smoothed maps are shown. D: dorsal, A: anterior, scale bars: 1 mm. The scales also apply to (B, C).

Spatial differentiation in cortical functional organization can provide insights into principles of local and global information processing. As a primary auditory field, AAF expresses a tonotopic gradient (Figs. 4A, S3A, S3B). Repetitive click train stimuli revealed distinct and non-homogenous spatial distribution patterns for the different temporal response measures embedded in the tonotopic map. Voronoi-Dirichlet tessellation maps (see Materials and Methods) of VS, FR, and CV of ISI for six different repetition rates are shown for spatially smoothed values (weighted least-squares linear regression model; Fig. 4B, 4C, 4D, for raw values, see Fig. S4A, S4B, S4C). The majority of sites had only moderate VS values to low repetition stimuli (blue to green polygons in Fig. 4B). Distinct neuron clusters with high VS (yellow to red polygons) emerged in restricted tonotopic regions (Fig. 4B). These clusters showed persistent and precise spike timing over a fairly wide range of repetition rates (6–22 Hz for Fig. 4B), suggesting the existence of spatially restricted cortical networks with high temporal population fidelity interleaved with regions of low temporal fidelity in the local neuronal population.

The spatial pattern of FR differed markedly from VS (Figs. 4C, S4B). Throughout the entire CF range, high FRs (red polygons) were seen for a range of repetition rates. However, sites with the highest FR substantially shifted with changes in repetition rate. This effect was less apparent for VS. To illustrate these activation shifts more clearly, high FR loci in smoothed maps, categorized as >0.75 of normalized peak activity, are shown in red (gray: sites with <0.75 of normalized FR, Fig. 4C). Even small changes in repetition rate activated a different cluster of cortical sites, i.e., comparable with a place code for low repetition rates.

The CV of ISIs also showed spatial clustering (Figs. 4D, S4C) with distinct regions of high (blue polygons) and low (red polygons) ISI precision that shift spatially with repetition rate although less clearly than for FR. The spatial changes of FR with increasing repetition rate appeared to be more widespread than for either VS or CV of ISI. Unlike FR, the two temporal precision measures showed spatially restricted regions of either high or low values that appeared largely invariant with repetition rate changes.

**Figure 5:**
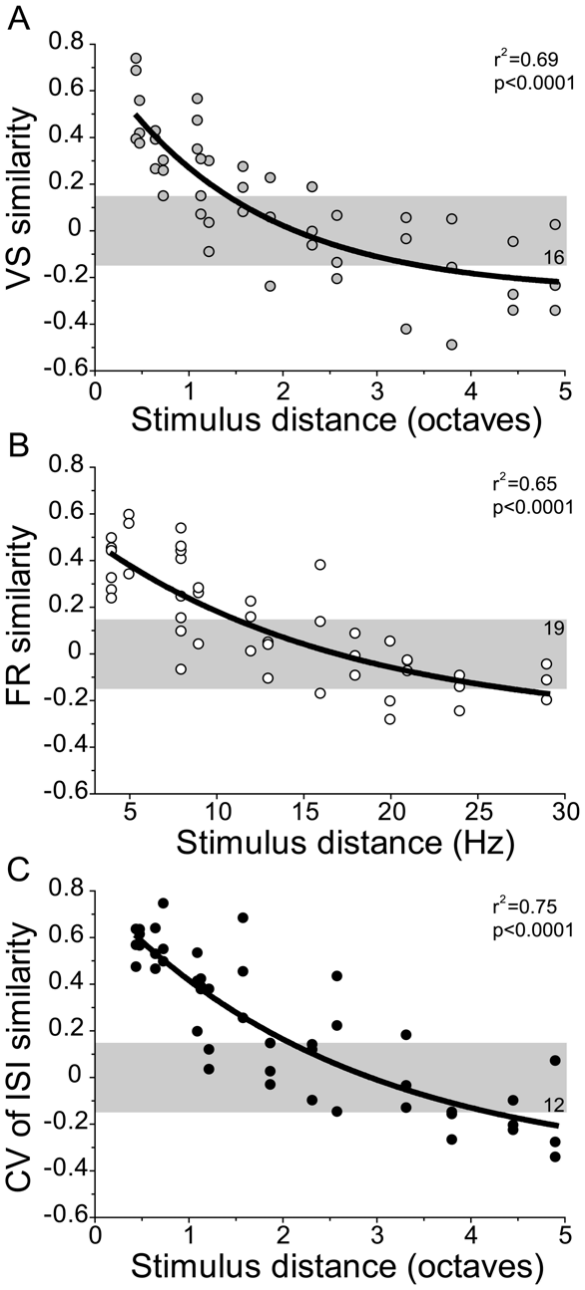
Map similarity for repetition rate differences. (A) Spatial cross-correlation values of raw VS values generated by repetition rates between 1 and 30Hz (see Fig. S4A) and plotted as a function of the logarithmic repetition rate difference for all three hemispheres. The solid line is a logarithmic fit. The gray area indicates non-significant correlation values (*n* = 16/45). (B) Cross-correlation values of raw FR generated by repetition rates between 1 and 30Hz (see Fig. S4B) and plotted as a function of the linear repetition rate difference. Non-significant correlations: *n* = 19/45. (C) Cross-correlation values of CV of ISI generated by repetition rates between 1 and 30Hz (see Fig. S4C) and plotted as a function of the logarithmic repetition rate difference. Non-significant correlations: *n* = 12/45.

Similarity analysis (spatial cross-correlation, see Materials and Methods) of the value distributions by cross-correlation as a function of repetition rate difference (Fig. 5A, 5B, 5C) showed that similarity reduction is proportional to the repetition rate difference for all three measures. The steepest decline in FR similarity was seen for small linear rate differences (Fig. 5B) with no significant correlations (−0.15<*r*<0.15) remaining for rate differences above 10–12 Hz. For VS and CV of ISI, the decline is proportional to the logarithm of the rate difference and steepest for 0.5 to 1 octave repetition rate differences (Fig. 5A, 5C) with a loss of correlation (−0.15<*r*<0.15) for rate differences above 1.5 to 2 octaves. Large repetition rate differences (>20Hz or >3.5 octaves, respectively) could show a weak anti-correlation between the spatial activation patterns. The logarithmic versus linear difference in the timing- and rate-based spatial pattern changes of click train responses suggests distinct divergence in the shapes of the corresponding RRTFs. This indicates differences among the encoding schemes and what is capture about the repetitive sounds.

Assessment of spatial organization in cortical fields requires rigorous statistical testing. We applied two approaches to determine the presence of spatial clustering for ten temporal and three spectral measures determined for the three hemispheres (Table 2; for spectral and response latency measures, see Ref. [30]). Spatial analysis for the combined additive or multiplicative information analyses was not pursued due to the analysis-based decrease in the number of valid recording sites.

**Table 2:** Spatial clustering statistics of cortical maps.

Hemisphere	111L	073L	073R	111L	073L	073R
**Statistics**		Global			Local	
**Measure**		Geary's C			Polygon Similarity	
**Polygon numbers**				130	76	70
CF	**1.44**	**1.47**	**1.47**	**0.86**	**0.92**	**0.90**
Q40	**1.26**	**1.17**	**1.13**	**0.29**	**0.34**	**0.30**
Threshold	**1.20**	**1.06**	**1.10**	**0.55**	**0.29**	**0.17**
Latency	**1.07**	**1.15**	*1.03*	**0.11**	*0.08*	**0.11**
VS info	*1.00*	*1.03*	**1.10**	**0.11**	**0.22**	**0.21**
FR info	**1.05**	*0.98*	**1.07**	**0.12**	*0.01*	*0.06*
ISI info	*1.03*	*0.98*	*1.01*	**0.26**	**0.13**	**0.14**
VS max	*0.99*	*1.03*	*1.02*	**0.15**	**0.17**	**0.10**
FR max	*0.99*	*0.99*	**1.04**	**0.28**	**0.12**	*0.09*
CV min	*0.99*	*1.03*	*1.01*	**0.15**	**0.12**	*0.09*
F1	*0.99*	*0.97*	*1.03*	**0.29**	**0.09**	**0.11**
F2	**1.04**	**1.05**	*1.03*	**0.16**	**0.30**	*0.09*
F5	**1.06**	*0.97*	**1.07**	**0.13**	*0.01*	*0.04*

Spatial autocorrelation analysis measured by Geary's C provides a global assessment of spatial organization versus random distribution. A stringent analysis of local similarity and parameter clustering was performed by determining average value differences of each polygon from its direct neighbors. Polygon similarity expresses the proportion of sites with significantly similar neighbors (range 0–1). Both global and local measures were validated by Monte-Carlo analysis. Bold: *p*<0.05; italic: not significant.

Spatial autocorrelation, validated with a Monte-Carlo analysis of randomized value assignments, was used to determine global trends of spatial organization (see Materials and Methods). Significant global organization was found for all three spectral parameters (CF, Q40 and response threshold; Table 2). Temporal response or information measures showed less reliable tendencies of global organization. Two hemispheres (111L and 073L) showed significant global organization for response latency and the temporal factor F2 (capturing VS information) and two hemispheres (111L and 073R) showed global spatial organization for FR information and F5 (also capturing FR information). No significant global organization was observed for ISI information, VS max, CV min, and F1 (aligned with ISI information and CV min). Temporal response parameters appeared to be less globally organized than spectral parameters.

**Figure 6:**
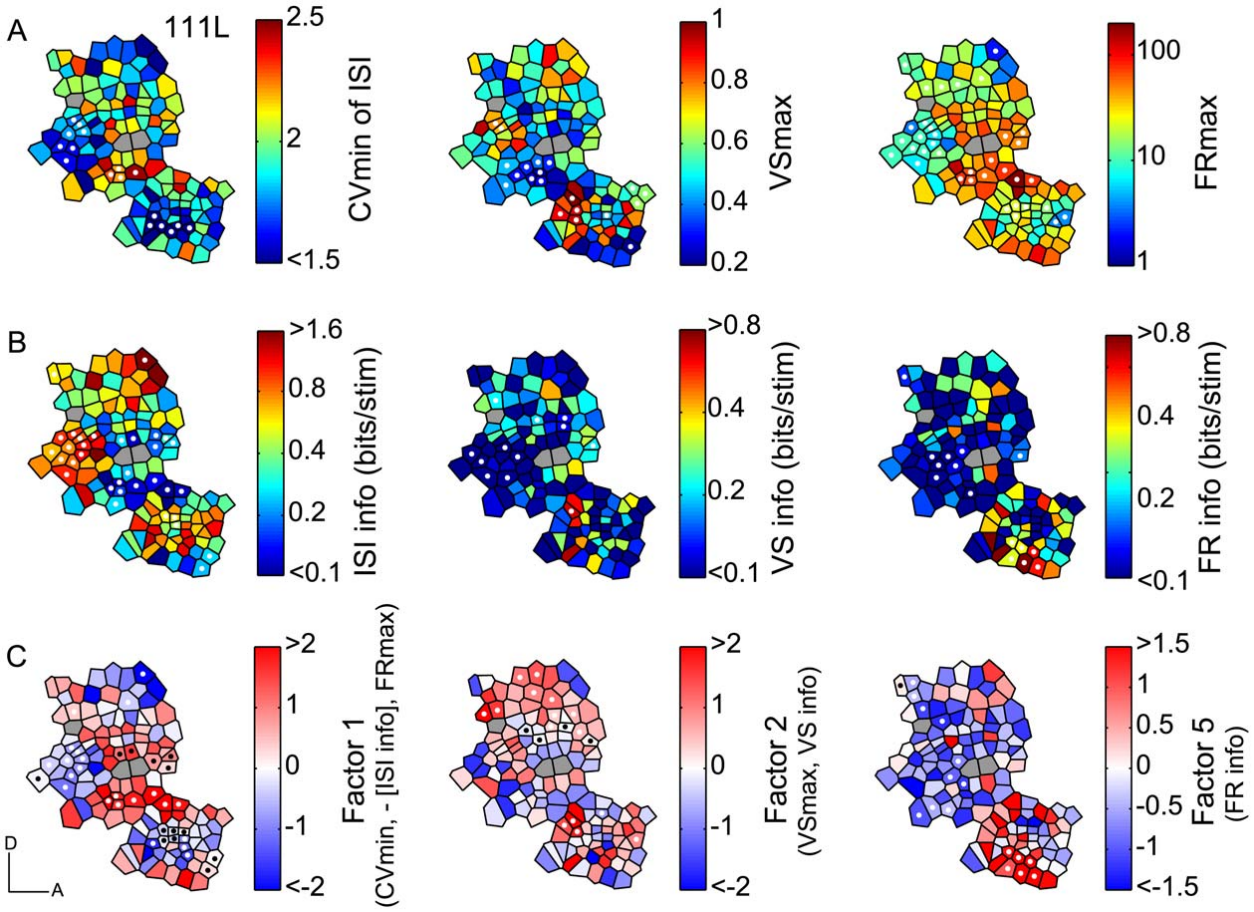
Spatial distributions of temporal response measures and mutual information. (A) Spatial distributions of CV min of ISI, VS max, and FR max for hemisphere 111L. Minimum or maximum value of the measures for any of the repetition rates is shown. White dots indicate polygons with statistically similar values as their direct neighbors (compared to random re-distribution of all neighbors, see Materials and Methods). Gray polygons indicate sites not available due to the four tested repetition rates, which also apply to (B, C). (B) Spatial distributions of mutual information values of ISI, VS, and FR based on repetition rate discrimination. (C) Spatial distribution of three temporal factors emerging from a principal component analysis of CV min, FR max, VS max, as well as the three corresponding information measures. Both white and black dots indicate polygons with statistically similar values as their direct neighbors.

**Figure 7:**
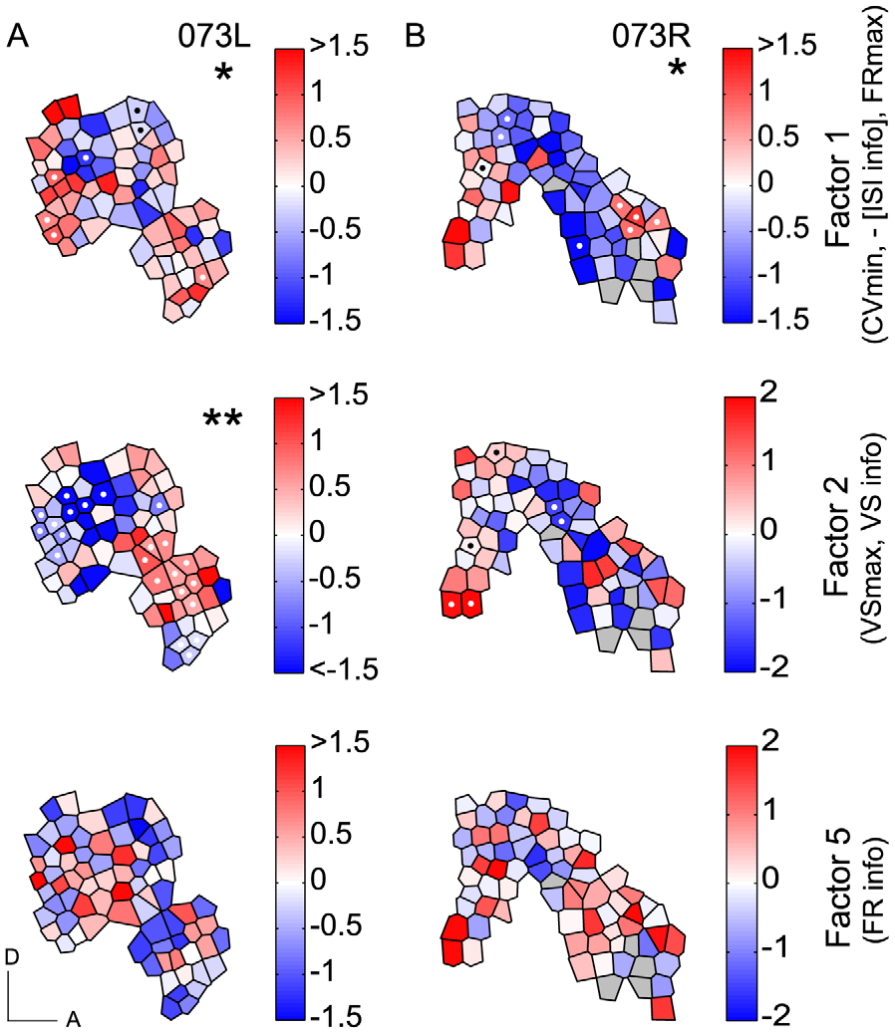
Spatial distributions of temporal response factors. (A) Spatial distribution of the magnitudes of three temporal factors based on a principal component analysis of CV min, FR max, VS max as well as the three corresponding information measures (hemisphere 073L). White and black dots indicate polygons with statistically similar values than their direct neighbors (compared to random re-distribution of all neighbors, see Materials and Methods). Significant local clustering: *: *p*<0.05; **: *p*<0.01. D: dorsal, A: anterior, scale bars: 1 mm. For the tonotopic gradient, see Figure S3. (B) Same as (A) for hemisphere 073R.

A non-significant Geary's C (see Materials and Methods) does not necessarily indicate absence of any spatial organization since spatial heterogeneity within a field leads to the possibility that global spatial auto-correlation may miss local organizations. Therefore, we applied a local analysis that tested the value similarity of each polygon with its direct spatial neighbors. The local similarity measure was validated by Monte-Carlo analysis (see Materials and Methods). The spatial distribution of nine temporal parameters for one hemisphere (111L) is shown in Figure 6. Only three maps showed global organization expressed by significant Geary's C (Table 2): FR information, and temporal factors F2 and F5. In contrast, local spatial organization was found for all 9 maps (Table 2), i.e., a statistically significant proportion of polygons had sufficient numbers of neighboring sites with similar values (Fig. 6, polygons with significant parameter clustering or neighborhood similarity, *p*<0.05, are marked by white and black dots). Each significant polygon could be considered as the center of a local, functionally similar cluster of neurons. The average spatial extent of such clusters, given the sampling density in these maps, was approximately 200–400 µm. Out of 27 maps constructed for temporal click rate parameters (3 hemispheres, 9 parameters), 20 maps exhibited significant local functional clustering. F1, the factor associated with ISI information, showed local clustering for all three hemispheres (Figs. 6C, 7), although global organization did not reach significance for any of them (Table 2). Both, local and global organization was evident for F2 in two hemispheres (111L and 073L) and for F5 in one hemisphere (111L). The average proportion of polygons with statistically significant neighborhood similarity for repetition rate response was generally <20% (Table 2). By contrast, spectral parameters showed a much higher proportion of polygons with functionally similar neighbors (Table 2). For CF, ∼90% of polygons had similar neighbors, and an average of ∼33% polygons showed highly similar neighbors for Q40 and response threshold.

**Figure 8:**
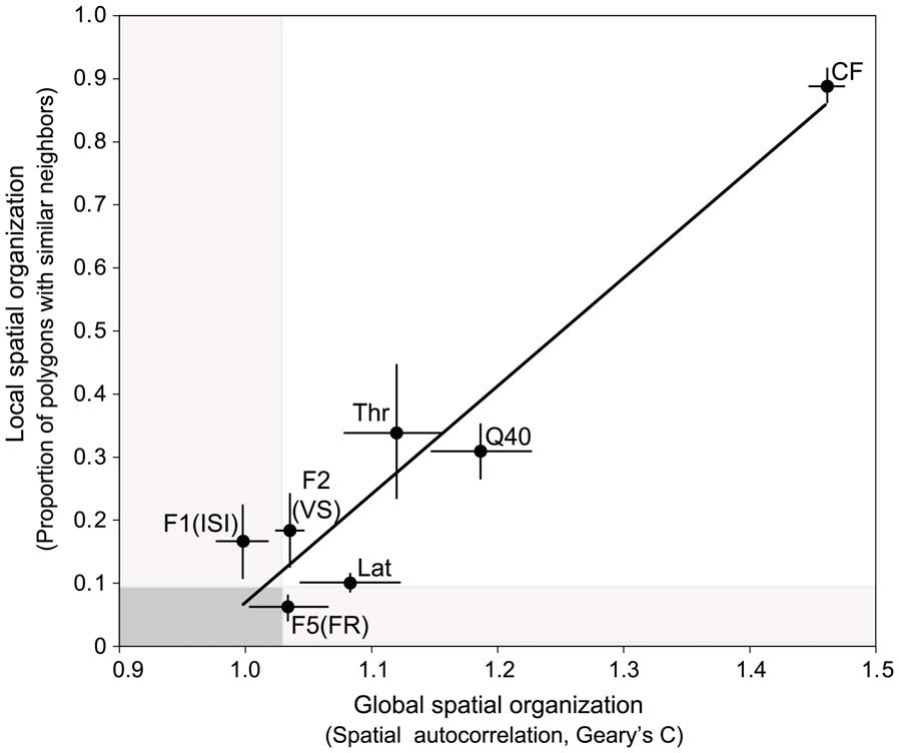
Local versus global spatial organization in AAF. A scatter plot of the mean proportion of polygons with high magnitude similarity to directly neighboring polygons (local spatial organization) versus a mean spatial autocorrelation measure (Geary's C; global spatial organization) for all three hemispheres (see Table 2). Error bars indicate standard error of the mean. Light gray shading indicates statistically non-significant regions for either measure. Dark gray area corresponds to value range that is not statistically significant for either local or global measures. A linear regression line is shown (*r^2^* = 0.92, *p*<0.001). CF = characteristic frequency; Q40 = frequency tuning curve bandwidth (at 40 dB above threshold)/CF; Lat = minimum response latency at CF; F1(ISI) = strongest temporal factor comprising CV min, ISI info, and FR max; F2(VS) = second strongest temporal factor comprising VS max and VS info F5(FR) = third strongest temporal factor comprising FR info.

A close relationship existed between the measures of local and global organization. Additionally, a hierarchy of the extent of spatial organization emerged across all tested parameters. The average global and spatial indicators of spectral and temporal spatial organization were highly correlated (*r^2^* = 0.92; *y* = 1.72*x*−1.65; Fig. 8). The highest degree of spatial organization in AAF was for CF, followed, in descending order, by spectral integration (Q40), and response sensitivity (threshold). Response latency and F2 (VS information) showed the most spatial organization among temporal parameters -both locally and globally- although clearly less than for the spectral parameters. F1 (ISI information) and F5 (FR information) revealed the least spatial organization with F1 showing only local clustering and F5 only global spatial trends. The main conclusion from the spatial analysis is that every investigated parameter can show some form of spatial organization, albeit the degree of spatial order can vary from highly organized pattern, with only shallow gradients extending over several millimeters of cortical space (e.g., CF), to lower order with scattered functional clusters of a few hundred µm diameter (e.g., temporal F1 and F5, see Figs. 6, 7).

## Discussion

Natural signals, in particular those used for communication, are characterized by low repetition rate or low frequency modulation. In this study, we demonstrated that the cortical neurons use multiple strategies to robustly process low repetition rates [7]–[10]. Low modulation rates dominate temporally encoded auditory cortical activity [6]. Neural coding of low repetition rates develops during AC maturation [43], [44] and in adulthood can be improved by behavioral training [45]–[47], experience, and hormonal manipulation [48]. Therefore, it is important to understand how low repetition rate sounds are encoded. Past studies focused on either VS or FR as largely alternative means for cortical encoding of slow repetitive sounds [6], [13]–[19]. Here we considered the contribution of ISIs to the encoding of low repetition rate sounds in AC.

### Repetition Rate Transfer Function Filters

The best click repetition rates for FR (∼29 Hz) in this study is in the same range as in a previous study using amplitude modulated signals in the barbiturate-anesthetized cat AAF (27 Hz, see Ref. [49]). For VS values, the current study (∼13 Hz) shows lower values than in the previous study (31 Hz, see Ref. [49]). These values in the current study are substantially higher than in a previous study using clicks in ketamine-anesthetized cat AAF [50]. The cause for the differences seen between the two studies may underlie difference in the sampling methods because similar anesthetic regimens were used.

Distributions of RRTF filter types can provide insight into temporal coding strategies. Several studies have described RRTF or modulation transfer function filter types [6], [14], [18], [19], [51] with the majority of recording sites in cat AAF (70–90%) revealing band-pass filtering property for VS and FR (see Ref. [6]). Despite differences in model systems (cat, old and new world monkeys), auditory cortical fields (AI, the anterior field, and others), recording conditions (anesthetized and awake), cortical layers (granular and supragranular layers), stimuli (click trains and amplitude modulated sounds), and classification criteria, the filter type distributions in the majority of those studies are in general agreement with the current study (70–80% were of band-pass type, data not shown).

Different RRTF filter shapes combined with different best repetition rate estimates may be suitable to code a wide range of repetition rates by utilizing temporal and/or rate coding strategies at the single neuron level [14], [16], [18], [19]. However, spike-timing precision and FR are not the only spike-train parameters that can reflect the nature of the stimulus. For visual and somatosensory cortical neurons, it has been shown that they are well equipped to decode stimulus-related information on the basis of relative spike timing and ISI duration [20], [52]. Our information analysis of auditory cortical neurons also demonstrates an advantage of interval timing over VS and FR in encoding and decoding of low stimulus repetition rates.

### Mutual Information Differences for Different Repetition Rate Codes

All three response measures (spike-time precision (VS), average FR, and ISI) provide information about the presented repetition rates. The amount of ISI information significantly exceeds that of either VS or FR alone. The highest ISI information values encountered here (Fig. 3) approach the theoretical value of ∼2.58 bits/stimulus (for consideration of discrimination between 6 repetition rates). However, the average ISI information remains clearly below the maximal value partially due to the use of multi-unit responses with overlapping responses from several neurons and noise contributions (see also Ref. [53]) as indicated by an information analysis restricted to intervals ≥10 ms. It is clear, however, that, for the average site, a substantial amount of information about the stimulus is being conveyed by other means.

While VS and FR provide fairly little information on their own, both parameters contribute to the CV of ISI and, consequently, to the ISI information content. VS max is positively correlated with ISI information. A high absolute spike-timing precision is advantageous for precisely encoding stimulus-based relative interval durations. FR magnitude is negatively and exponentially correlated to the ISI information. This is not unexpected since higher FRs will result in a higher probability of shorter ISIs that are independent of the stimulus-driven interval statistics, especially in cases of multi-unit recordings as employed here. Such an inverse relationship between FR magnitude and the amount of MI is also found in single neurons of the cat mid-brain [54] and in visual neural transformation from retina to thalamus in the macaque monkey [55]. In the former study, neurons with small FR magnitude also showed high information content per spike and high feature selectivity. Such feature selectivity by small number of spikes (sparse coding) is found for odor coding in the insect mushroom body [56] and mammalian olfactory cortex [57], for song syllable sequence in the song bird premotor area [58], and for constructing an acoustic image by multiple delays in the echolocating bat AI [59]. Therefore, a close relationship between FR magnitude and ISI information may be a basis of neural coding of communication calls at the primary cortical level. There are several potential benefits to maintaining a temporal code of repetition information at the level of AAF. Temporal information may be more easily transmitted to the following stations that can read out the information via converging projections and precise coincident inputs. Energy consumption may be lower for a low-rate interval code than for an average rate code. Stimulus-locked temporal codes may provide useful information about a task or stimulus that may not be necessary for single discrimination or detection tasks and could be accounted for by rate measures alone as demonstrated in the detection of vibratory stimuli [52].

The finding that the information related to the three temporal codes project onto orthogonal factors points to some non-redundancy in the different periodicity representation schemes. This is also expressed in the dissimilar spatial distribution pattern and their distinctions in repetition rate dependence. Availability of different encoding schemes may have advantages for signal processing under different conditions and adverse circumstances such as low signal-to-noise ratios, reverberation, variations in sound intensity, or the presence of multiple sound sources that may affect the three codes in different ways. The possibility that different codes, employed concurrently, can provide complementary information has already been demonstrated for natural sounds [53]. A study of the neural ensemble code for stimulus periodicity in the range of the fundamental frequency of vocalizations also demonstrated that a joint code of rate and timing parameters provide more information than either code alone [60]. The observation that the combined FR and ISI information reflects an increased amount of repetition information confirms the representation of non-redundant information by rate and temporal codes also for low repetition rates in AAF.

It should be noted that the three coding aspects discussed here for repetition rate discrimination do not provide a complete picture of low modulation frequency analysis. Recent studies have pointed out additional means to detect and discriminate the waveform shape of slow modulations, relying on more complete analyses of the evoked spike patterns, and their relationship to rhythmic activity [18], [53], [61], [62].

### Spatial Organization of Repetition Rate Coding

Stimulus information is distributed across a wide range of cortical neuron types, laminae, and areas. Knowledge of the spatial layout of information processing is important because it can provide crucial insights into the local functional tasks and algorithms [25], [63]. Several aspects of spatial organization and variability emerged.

The two temporal response measures and FR show different kinds of spatial variations with repetition rate changes. Nearly stimulus-independent sub-regions were observed for VS and CV of ISI. These ‘modules’ with locally confined variations are in contrast to spatial FR patterns that shift over a wider area in a stimulus-dependent manner, more compatible with a rate/place code. This difference in spatial behavior for timing and rate codes is also expressed by a scaling difference. The largest changes in FR map similarity are observed for small, linear repetition rate differences in contrast to small changes on a logarithmic scale for temporal maps. These differences in type and stimulus dependence of the spatial distributions indicate a degree of independence of time and rate codes for periodicity analysis. The factor analysis supports the notion that the three stimulus repetition codes considered here operate somewhat, although not completely, separately. Differences in the spatial behaviors of spike-timing precision, rate, and interval codes as a function of repetition rate can be interpreted as evidence for multiple, concurrent processing streams (or streamlets) embedded within a cortical area.

Stimulus-tolerant spatial features (Figs. 4B, 4D, S4A, S4C) likely reflect specialized and confined anatomical networks [25], [63], [64] that can support a stable connectional framework for task-specific processing. Separate neuron clusters for either precise or only moderately synchronized spike timing can be a consequence of convergent thalamocortical projection to AAF [28], [30] and local cortical circuits properties [13] that may be expressions of structurally and functionally distinguishable components of larger, more generally definable processing and projection schemes such as the ‘what’ and ‘where’ streams. Reading out information from stimulus-dependent maps would require broad-range connections, while reading out information from a locally stimulus-independent map could be done through local connections alone.

### Hierarchical Spatial Order in Auditory Cortex

A novel, quantitative spatial analysis of cortical maps revealed that local clustering of similar functional properties is a general feature of all parameters considered here. Local clustering exceeding the expectations from random parameter distributions were encountered for spectral and temporal parameters in ∼80% of the maps. Failure to observe significant clustering in the remaining 20% may be a consequence of the sampling density and the ratio of circumference-to-area of the mapped region with reduced statistical power in cases of high ratios (e.g., hemisphere 073R; Fig. 7).

The proportion of sites that are surrounded by sites with similar properties can vary over a wide range from <10%, for some temporal parameters, to >90% for frequency preference (i.e., CF). Maps with low clustering proportions often have only few, isolated sites with similar surroundings. With increase in the overall clustering proportion confluence of individual clusters to larger modules is observed and, finally, large-scale aggregates, such as the tonotopic organization, are seen for maps with high clustering proportion. This range or hierarchy of spatial order in cortical maps is confirmed by the analysis of global spatial organization through spatial autocorrelation and can now be quantified (e.g., Geary's C) and compared across different areas and modalities. It should be noted that the current analysis methods do not require or rely on the notion of local functional gradients that in previous studies have been the dominant feature in assessing functional topography (e.g., see Ref. [26], [63]).

Anatomical studies of AC have revealed that all extrinsic areal connections, whether tonotopic, non-tonotopic, multisensory, or limbic, show a high degree of connectional topography [63], [64]. Local topographies in convergent inputs create distinct conditions for functional processing and it is, thus, not surprising to see topographic principles expressed by essentially all considered functional aspects in AAF. Similar spatial order is conceivably present in areas outside the core areas although it is currently not clear where they fall along the continuum of a spatial order hierarchy and what the functional parameters are that may be organized in such a way.

### Methodological Considerations

Recording conditions used in this study influenced all three measures of VS, FR, and ISIs. Our data were predominantly based on multi-unit recordings since one of the goals was to elucidate the spatial distribution pattern of the different response measures. There are separate loci with either low or high VS. Neuronal clusters with low VS may arise from single neurons within a recorded cluster with precise spike timing at different phases of stimulus period (thus, resulting in only moderate spike timing in multi-units) and/or individually less precise spike timing. Neuronal clusters with high VS are loci of very precise and highly synchronized spike timing that reflect a tight network organization.

FR might also be influenced by the number of neurons in the recorded clusters. Because FR information is not related to FR magnitude, multi-unit recordings do not appear to strongly influence the obtained FR information. On the other hand, the amount of ISI information is negatively correlated with FR magnitude. Therefore, it cannot be determined whether low ISI information is associated with the number of neurons in a recorded cluster and/or the number of spikes.

Finally, high ISI variability may depend on the number of neurons within a cluster, synchronized spike timing among the neurons, and interval variability within single neurons. Low fidelity in any of these aspects may dominate sites carrying low ISI information. Comparisons of single- and multi-unit recordings made for periodic click trains revealed no systematic differences [50]. Furthermore, temporal response properties are mostly independent of CF, thus local disparity in frequency tuning is not likely to strongly effect the temporal response properties [50]. However, multi-unit responses may not simply represent a cluster of single-unit properties. Therefore, single-unit recordings will be necessary in both acute and awake preparations to provide a fuller understanding. The current study provides a more general framework for such future investigations.

### Comparative Aspects for Processing of Speech and Communication Sounds

Recent studies using functional magnetic resonance imaging or positron emission tomography in humans and macaques suggested that the superior-temporal plane is specific to human speech or macaque species-specific calls over non-specific calls or other sounds [65]–[67]. These fields are located anterior to the primary core fields, and may be a part of an anterior auditory ‘what’ pathway [34].

The anterior field of AC is found in many different animal models (for review, see Ref. [30]). Several studies of neural processing of repetition rates or amplitude modulated sounds have indicated that AAF may show higher temporal fidelity than other cortical fields [6], [35], [49]. Furthermore, behavioral experiments have suggested that AAF may be a suitable area to study the neural processing of temporal sound aspects and, more generally, may be part of system focused on object-based or ‘what’ properties of the auditory environment [32]. Recently, Bendor and Wang [19] proposed that the rostral field (R) of marmoset AC dominantly uses a rate code for a particular repetition-rate range (10–45 Hz). While anatomical locations (position relative to AI) of cat AAF and marmoset R are similar, it is not known whether these two fields share similar physiological and anatomical properties.

Overall, the findings suggest local processing specialization within an early cortical station of the ‘what’ pathway, suggesting the presence of subdivisions within more global processing streams. The observation that an interval code allows more discrimination ability of periodicity information than codes either based solely on temporal precision or mean FR may provide a convenient window to assess mechanisms and local tasks implemented in an anterior auditory pathway that emphasizes temporal aspects of sound processing. The observation that local spatial organization, in form of functional mini-modules, may be ubiquitous can guide future attempts to reconcile functional and structural organizational principles [68] within and across different processing streams.

## Materials and Methods

### Surgery and Animal Preparation

Experiments were conducted on three hemispheres (two left and one right hemispheres) of two adult female cats. All protocols were approved by the University of California San Francisco Committee on Animal Research in accordance with federal guidelines for care and use of animals in research. Animals were sedated by intramuscular injections of a mixture of ketamine (22 mg/kg) and acepromazine (0.11 mg/kg). After venous cannulation, sodium pentobarbital (15–30 mg/kg) was administered and supplemented as needed throughout the surgical procedure. Following tracheotomy, a craniotomy was performed to expose the ectosylvian gyrus. The dura mater was partially removed, and the cortical surface was covered with viscous silicone oil. Before commencing the electrophysiological recordings, sodium pentobarbital anesthesia was replaced with a continuous intravenous infusion of a mixture of ketamine (2–10 mg/kg/h) and diazepam (0.05–0.2 mg/kg/h) in lactated Ringers (1–3 ml/kg/h). To prevent edema and mucus secretion, dexamethasone (1.2 mg/kg, S.C.) and atropine sulfate (0.04 mg/kg, S.C.) were administered every 12 hours. Since recordings lasted for three to four days, an antibiotic (cephalosporin, 11 mg/kg, I.V.) was administrated to prevent wound infection. Body temperature was monitored and maintained by a water heating pad at 37±1°C. Electrocardiogram and respiration rate were monitored continuously during surgery and recording procedures.

### Acoustic Stimulus and Extracellular Recordings

Experiments were conducted in a double-walled, anechoic chamber (Industrial Acoustics, Bronx, NY). Stimuli were delivered by a STAX-54 headphone through a sealed tube into the acoustic meatus contralateral to the studied hemisphere. The system frequency transfer function was flat (±6 dB) up to 14 kHz and rolled off 10 dB/octave at higher frequencies.

Two different stimuli, pure tone bursts and click trains, were presented for measuring frequency response areas (FRAs) and RRTFs, respectively. Pure tone stimuli of 50ms duration (including 3-ms linear rise and fall time) were generated at intervals of 400–750 ms by a microprocessor (TMS32010, 16 bits resolution and 120 kHz digital-to-analog sampling rate). FRAs were mapped by presenting 675 pseudo-randomized tone bursts at 45 different frequencies (3–5 octave range) and 15 sound levels (70 dB range in 5 dB steps). For RRTFs, click trains (monopolar, rectangular pulses of 200 µs duration; 500 ms train duration) were systematically presented 15 times for repetition rates from 1 to 38 Hz (1, 6, 10, 14, 18, 22, 26, 30, 34, and 38 Hz) at sound levels of 82–102 dB SPL (peak equivalent). The relatively high levels were applied to enhance synchrony among the multi-unit responses. For sites with non-monotonic rate-level functions values at the lower end of the range were used. For some recording sites, higher repetition rates were presented (up to 250 Hz).

Parylene- or epoxylite-coated tungsten microelectrodes (Micro Probes, Potomac, MD or Frederic Haer & Co., Bowdoinham, ME) with 0.5–4 MΩ impedance at 1 kHz were used for multi-unit recordings. Single or double microelectrodes were advanced perpendicular to the cortical surface with a hydraulic microdrive (David Kopf Instruments, Tujunga, CA). A video picture of the cortical surface was captured and digitized with a CCD digital camera (Cohu, San Diego, CA). Each recording site was marked on the digitized picture using Canvas software (Deneva, Miami, FL). The marked sites were used to reconstruct tessellation maps of the recording area (see below). Neuronal activity was obtained in thalamocortical recipient layers [69]. Action potentials were amplified and band-pass filtered (0.3–10 kHz; World Precision Instruments, Sarasota, FL, and Axon Instruments, Union City, CA), fed to an oscilloscope, and isolated from background noise with a time/amplitude window discriminator (BAK Electronic, Mount Airy, MD). For FRAs and RRTFs, spikes occurring in the first 50 ms or 550ms, respectively, after stimulus onset were recorded at 10 or 100 µs resolution for the analyses.

### Data Analysis

Data were analyzed using the MATLAB (Mathwork, Natick, MA) platform. StatView (SAS Institute, Cary, NC) was used for statistical analysis.

Spectral receptive field parameters such as CF, minimum threshold, quality factors, and response latency were measured [30]. Threshold was defined as minimum excitatory SPL, and estimated at 5 dB resolution. CF was defined as the frequency at which a single neuron or neuron cluster produced sound-evoked spikes at threshold sound level. Spectral bandwidths were calculated as CF divided by excitatory bandwidth at 40 dB (Q40) above threshold; the higher the Q-value, the more sharply tuned are the neurons. Latency was determined as the minimum value in the averaged latency-level function at CF and the two adjacent test-frequencies (CF 1/15 to 1/9 octaves). Results for spectral receptive field parameter distributions in AAF were presented elsewhere [30].

For RRTFs, spike occurrence to the first click was discarded except for the 1 Hz stimulus since it does not contribute to repetition information. Spikes were counted from the second click onset to 550 ms after the first click onset (for 1 Hz stimulus, spikes occurring between the first click onset and 550 ms were used). VS and FR were used to measure temporal following activity [6], [50]. VS measures how well spikes are synchronized to the clicks relative to the duration of the repetition period:
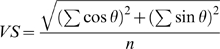


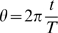
where *n* is the total number of spikes, *t* is time of spike occurrence, and *T* is the inter-click interval [70]. Significance of synchronization was examined by a Rayleigh test (*p*<0.001). Bin width was 1 ms. Repetition rate tuning curves were constructed without smoothing across different repetition rates. Best repetition rate was defined as that a repetition rate that evoked the largest response strength for VS or FR. RRTF tuning curves for VS and FR were classified into three filter types. A band-pass filter was assigned when the response peak was flanked by troughs in which the responses drop <75% of the peak [71]. If one of the response troughs did not reach the criterion, then RRTFs were considered to be either a low- or high-pass filter. Although most recordings were made from multi-units, past studies have shown that single- and multi-unit recordings share similar RRTFs or modulation transfer functions [50], [72], [73].

ISIs between two consecutive spikes were measured in the time window of 550 ms with a bin width of 1 ms for each trial and accumulated across all 15 trials. Spike train irregularity in ISIs was estimate based on the CV that was defined as the standard deviation of ISIs divided by the mean of ISIs.

### Voronoi-Dirichlet Tessellation Map

To reconstruct the spatial distribution of receptive field or temporal parameters across the cortical surface, tessellation maps were calculated by Voronoi-Dirichlet tessellation [74]. The polygon surrounding each electrode penetration in the tessellation map characterizes the area assigned to the functional parameter at the recording site. Borders between neighboring polygons were determined from the midpoints of a straight line between adjacent recording points. The value of each receptive field or temporal parameter in the cortical surface map is illustrated by color code.

### Mutual Information Analysis

The MI of the repetition rate carried in the FR was computed based on 15 presentations of the same set of repetition rates. MI analyses were limited to six different repetition rates (1, 6, 10, 14, 22, and 30 Hz for which we obtained data sets for a majority of recording sites). MI between repetition rate *f* and firing rate *fr* is given by 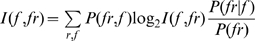. In our case, all repetition rates *f* were presented the same number of times, so that
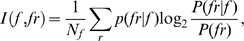
where *N_f_* = 6 was the number of different repetition rates. To account for the fact that MI is positively biased [75], [76], the values were linearly extrapolated to infinite dataset limit (i.e., number of repetitions; not to the limit of infinite word length). Extrapolation was done by removing different sets of one, two, three, or four presentations at a time. The final value and its standard deviation was obtained as a result of a linear fit in *1/N_f_*, each repeated 15 times for different combinations of dropped presentations.

MI between repetition rate and VS was evaluated similarly. VS values were calculated for each stimulus presentation to form distributions of VS values associated with each stimulus periodicity. The MI conveyed by the VS code quantifies how well these distributions (and thus stimulus repetition rates) can be distinguished from each other. Non-significant VS measures were assigned a MI of zero bits/stimulus (Rayleigh test, *p*>0.001). In the case of information carried by ISIs, the distribution of ISIs *P*(*isi*|*f*) was computed for each stimulus repetition rate *f* and averaged across repeated stimulus presentations. These information values were then also extrapolated to the infinite dataset size, according to procedures described above.

Additive information values (Code_(x)_ + Code_(y)_ in Fig. 2B) represent the sum of information values computed for each pattern of neural responses separately, with separate extrapolation to infinite dataset size. Joint information values (Code_(x)_ × Code_(y)_ in Fig. 2B) were computed based on joint probabilities of two measures of neural responses, such as VS and FR (Fig. 2B, white bars); extrapolation to infinite dataset size in this case was based on recomputation of these joint probabilities from fractions of the data, and then using a linear extrapolation with respect to the inverse of the dataset size to find the value for infinite dataset size.

### Spatial Organization Analysis

The existence of spatial organization for experimental variables was established using two complementary approaches. Spatial autocorrelation, a measure of redundancy, was used to estimate global spatial organization by calculating Geary's C coefficient [77]. C values are based on value differences between pairs of observations and can vary between 0, indicating perfect positive spatial correlation (high spatial uniformity, maximal neighbor similarity), and 2, indicating negative spatial correlation (maximal dispersion, high value contrast between neighbors). Random spatial distribution (the null-hypothesis) results in a C value of 1. In a Monte-Carlo analysis, the statistical significance of the experimental C value was derived from the C-value distribution of 10,000 randomly redistributed map versions.

Local spatial organization was assessed through the value similarity between each polygon and its nearest neighbors. Statistically significant similarity between a polygon and its direct neighbors was determined by comparison with 10,000 redistributions of the neighboring polygon values. The number of significant polygons in a given experimental map was compared to the number of significant polygons in 1,000 randomized maps. The number of significant polygons estimates the proportion of local parameter clusters. Neither of the two tests takes into account where in the map local or global similarities are situated. However, the larger the number of local clusters, the higher is the probability of a confluence of them, increasing global organization and, thus, spatial autocorrelation.

## Supporting Information

Figure S1 Two examples of RRTFs for VS and FR. (A, B) Poststimulus time histograms for two different sites. Response strength was normalized to the maximum responses at 1 Hz. (C, D) RRTF tuning curves for the same two sites. VS and FR are illustrated by magenta and cyan lines, respectively, and data points are fit by a polynomial cubic spline for illustration. Filled circles are significant VS values by a Rayleigh test (p<0.001). Site identification and CF are shown in (C, D). Gray areas are the repetition rate range for the focus of our study. (A, C) Moderate VS site. FR showed band-pass property with high best repetition rate. VS information: 0.19 bits/stimulus. FR information: 0.33 bits/stimulus. ISI (1 ms) information: 0.34 bits/stimulus. ISI (10 ms) information: 0.74 bits/stimulus. (B, D) Low VS site. FR increased with increasing repetition rates (high-pass property). VS information: 0.08 bits/stimulus. FR information: 0 bits/stimulus. ISI (1 ms) information: 0.58 bits/stimulus. ISI (10 ms) information: 1.17 bits/stimulus.
(0.43 MB TIF)

Figure S2 An example of ISI histograms for six different repetition rates from one site. There is no additional peak corresponding to the period of the stimulus repetition rates. This site is a less common example. VS information: 0.01 bits/stimulus. FR information: 0.02 bits/stimulus. ISI (1 ms) information: 0.15 bits/stimulus. ISI (10 ms) information: 0.45 bits/stimulus.
(0.08 MB TIF)

Figure S3 Smoothed tonotopic gradient and approximate position of AAF. (A) Hemisphere 073L. (B) Hemisphere 073R. Scale bars: 1 mm. See Figure 4's legend for further explanation.
(0.19 MB TIF)

Figure S4 Spatial distributions of population response (without smoothing) to different repetition rates (hemisphere 111L). Spatial representation of raw VS (A), normalized FR (B), and raw CV of ISI (C) as a function of different repetition rates. Scale bars: 1 mm. See Figure 4's legend for further explanation.
(0.86 MB TIF)
