# Supplementary figures and images for: Encoding of Temporal Information by Timing, Rate, and Place in Cat Auditory Cortex
Source: PLoS One. 2010 Jul 19;5(7):e11531. doi: 10.1371/journal.pone.0011531 (PMC2906504; doi:10.1371/journal.pone.0011531)

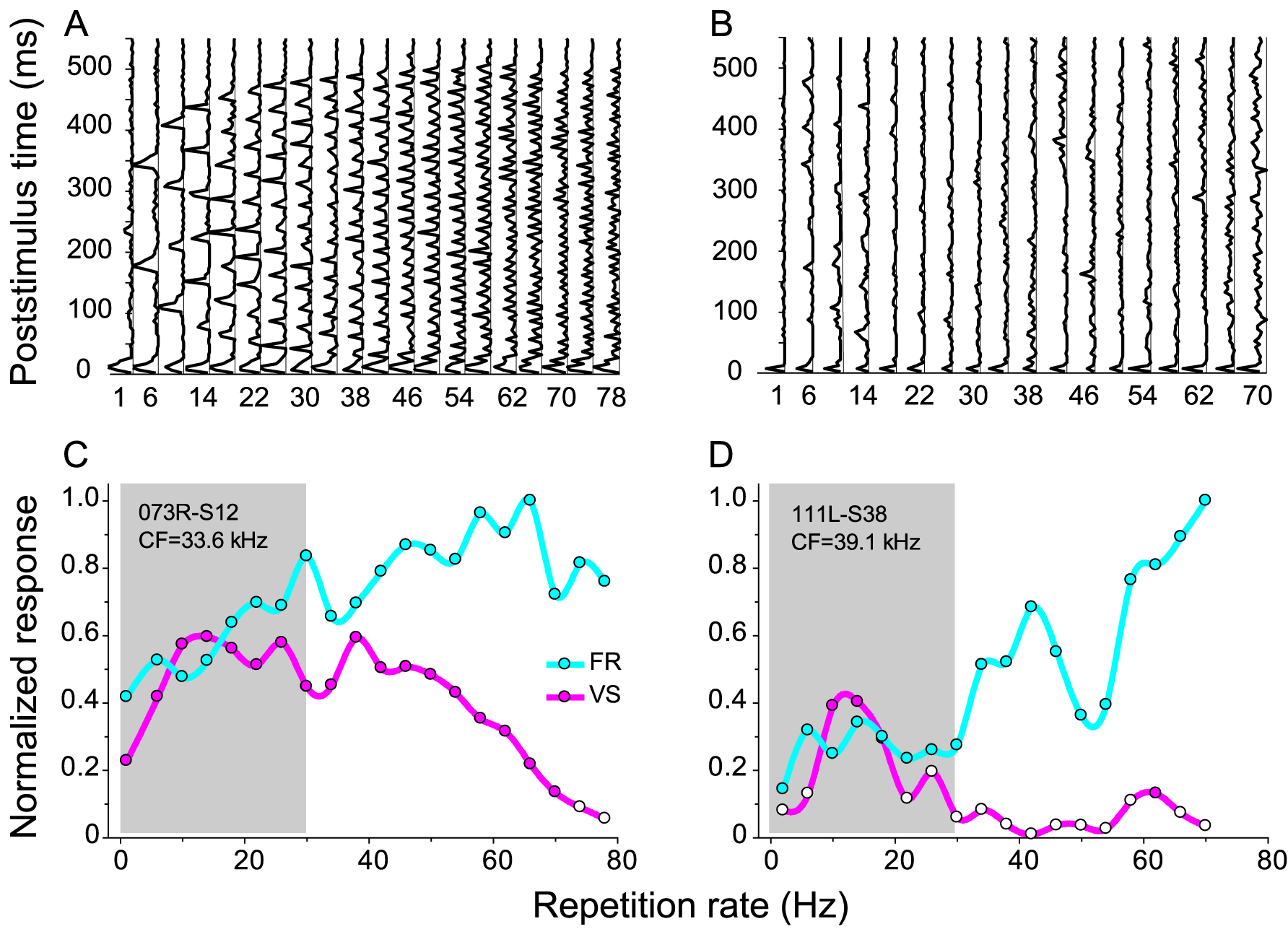

Supplement: Figure S1 — Two examples of RRTFs for VS and FR. (A, B) Poststimulus time histograms for two different sites. Response strength was normalized to the maximum responses at 1 Hz. (C, D) RRTF tuning curves for the same two sites. VS and FR are illustrated by magenta and cyan lines, respectively, and data points are fit by a polynomial cubic spline for illustration. Filled circles are significant VS values by a Rayleigh test (p<0.001). Site identification and CF are shown in (C, D). Gray areas are the repetition rate range for the focus of our study. (A, C) Moderate VS site. FR showed band-pass property with high best repetition rate. VS information: 0.19 bits/stimulus. FR information: 0.33 bits/stimulus. ISI (1 ms) information: 0.34 bits/stimulus. ISI (10 ms) information: 0.74 bits/stimulus. (B, D) Low VS site. FR increased with increasing repetition rates (high-pass property). VS information: 0.08 bits/stimulus. FR information: 0 bits/stimulus. ISI (1 ms) information: 0.58 bits/stimulus. ISI (10 ms) information: 1.17 bits/stimulus. (0.43 MB TIF) [file pone.0011531.s001.tif]

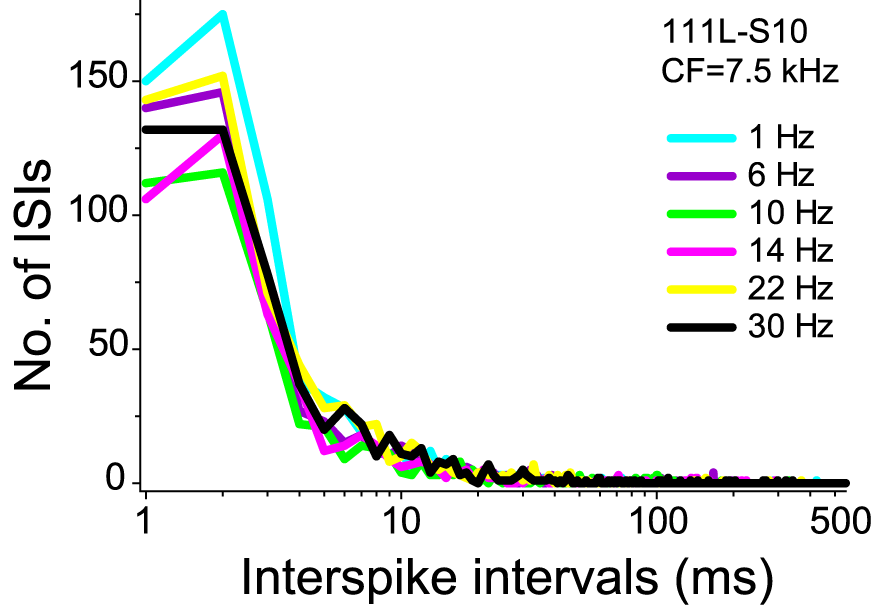

Supplement: Figure S2 — An example of ISI histograms for six different repetition rates from one site. There is no additional peak corresponding to the period of the stimulus repetition rates. This site is a less common example. VS information: 0.01 bits/stimulus. FR information: 0.02 bits/stimulus. ISI (1 ms) information: 0.15 bits/stimulus. ISI (10 ms) information: 0.45 bits/stimulus. (0.08 MB TIF) [file pone.0011531.s002.tif]

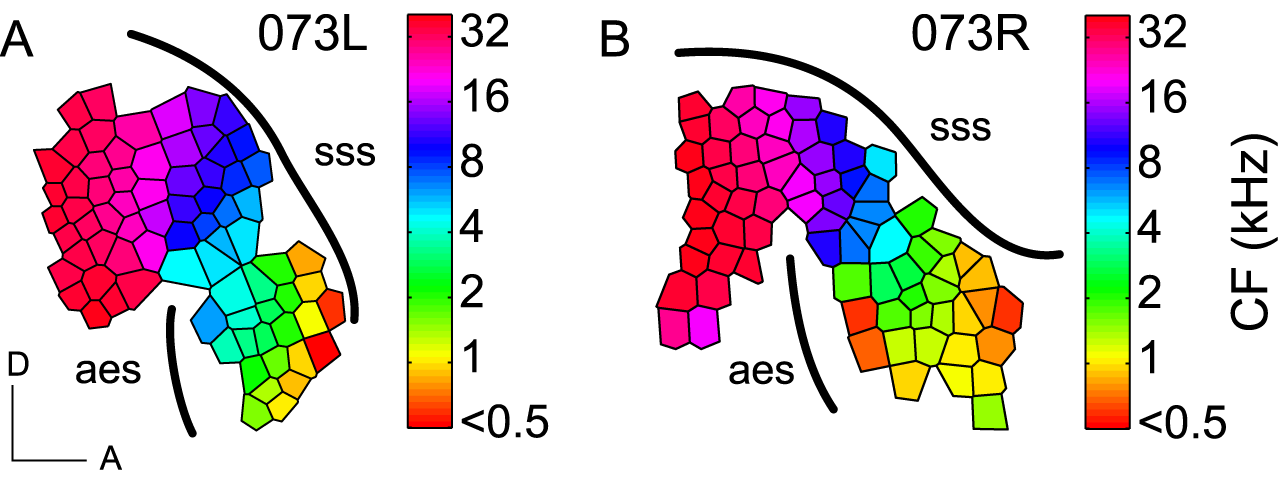

Supplement: Figure S3 — Smoothed tonotopic gradient and approximate position of AAF. (A) Hemisphere 073L. (B) Hemisphere 073R. Scale bars: 1 mm. See Figure 4's legend for further explanation. (0.19 MB TIF) [file pone.0011531.s003.tif]

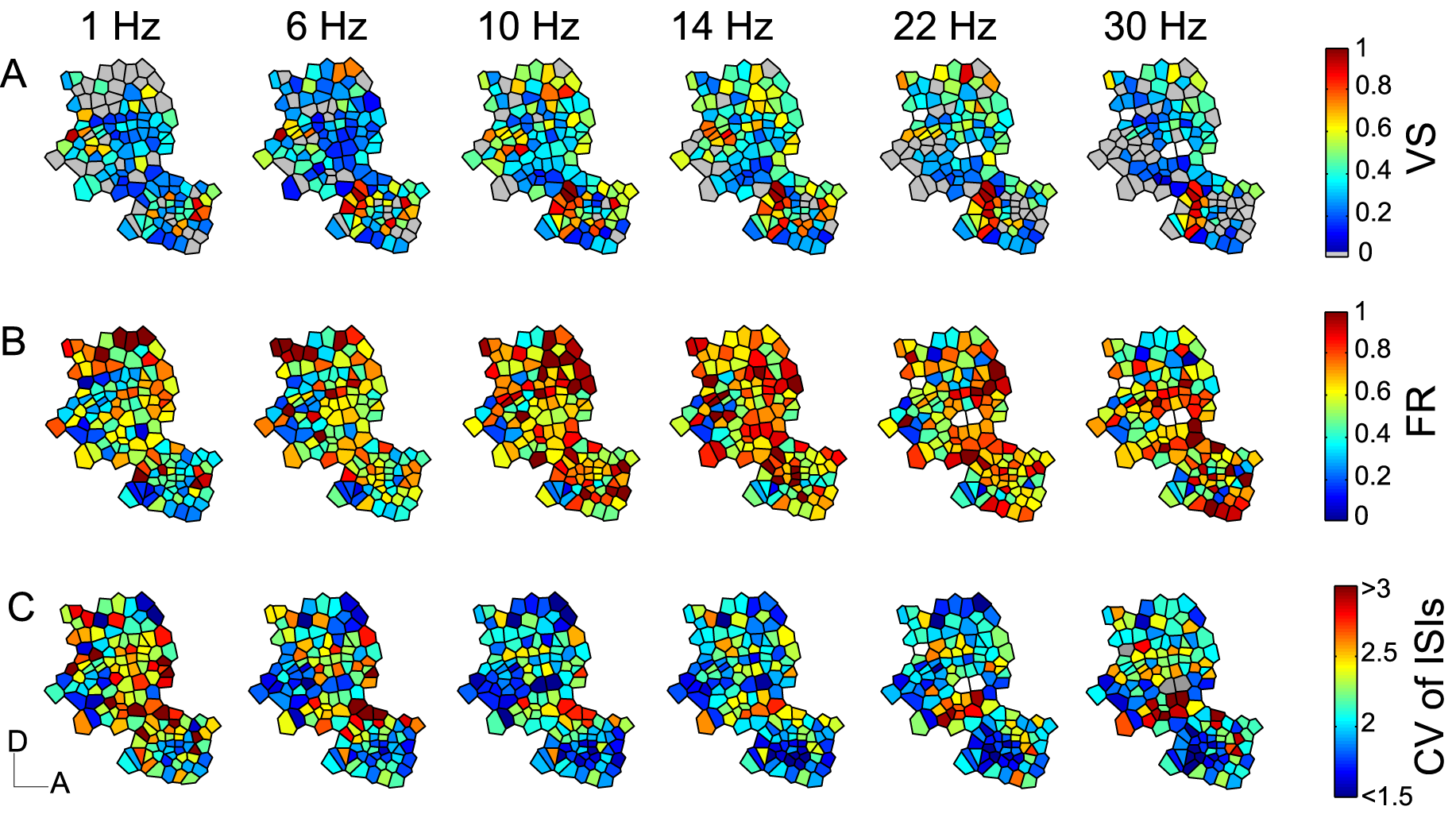

Supplement: Figure S4 — Spatial distributions of population response (without smoothing) to different repetition rates (hemisphere 111L). Spatial representation of raw VS (A), normalized FR (B), and raw CV of ISI (C) as a function of different repetition rates. Scale bars: 1 mm. See Figure 4's legend for further explanation. (0.86 MB TIF) [file pone.0011531.s004.tif]
